# Supporting self-management after attending a structured education programme: a qualitative longitudinal investigation of type 1 diabetes patients’ experiences and views

**DOI:** 10.1186/1471-2458-12-652

**Published:** 2012-08-14

**Authors:** David Rankin, Debbie D Cooke, Jackie Elliott, Simon R Heller, Julia Lawton

**Affiliations:** 1 Centre for Population Health Sciences, University of Edinburgh, Edinburgh, EH8 9AG, UK; 2 Epidemiology & Public Health, University College London, London, WC1E 6BT, UK; 3 Academic Unit of Diabetes, Endocrinology and Metabolism, University of Sheffield, Sheffield, S10 2JF, UK

## Abstract

**Background:**

Structured education programmes for patients with diabetes and other chronic conditions are being widely adopted. However, follow-up studies suggest that course graduates may struggle to sustain the self-care practices taught on their courses over time. This study explored the support needs of patients with type 1 diabetes after attending a structured education programme promoting an empowerment approach and training in use of flexible intensive insulin therapy, a regimen now widely advocated and used to manage this condition. The objective was to inform future support offered to course graduates.

**Methods:**

Repeat, in-depth interviews with 30 type 1 diabetes patients after attending Dose Adjustment for Normal Eating (DAFNE) courses in the UK, and six and 12 months later. Data were analysed using an inductive, thematic approach.

**Results:**

While the flexible intensive insulin treatment approach taught on DAFNE courses was seen as a logical and effective way of managing one’s diabetes, it was also considered more technically complex than other insulin regimens. To sustain effective disease self-management using flexible intensive insulin treatment over time, patients often expected, and needed, on-going input and support from health care professionals trained in the approach. This included: help determining insulin dose adjustments; reassurance; and, opportunities to trouble-shoot issues of concern. While some benefits were identified to receiving follow-up support in a group setting, most patients stated a preference or need for tailored and individualised support from appropriately-trained clinicians, accessible on an ‘as and when needed’ basis.

**Conclusions:**

Our findings highlight potential limitations to group-based forms of follow-up support for sustaining diabetes self-management. To maintain the clinical benefits of structured education for patients with type 1 diabetes over time, course graduates may benefit from and prefer ongoing, one-to-one support from health care professionals trained in the programme’s practices and principles. This support should be tailored and personalised to reflect patients’ specific and unique experiences of applying their education and training in the context of their everyday lives, and could be the subject of future research.

## Background

Type 1 diabetes is a long-term, chronic condition with incidence rates increasing at approximately 3% per year in countries around the world [1,2]. People with type 1 diabetes depend on regular insulin injections and must adhere to multiple self-care tasks to optimize glycaemic control; however, many patients struggle to self-manage their diabetes effectively [3,4]. If not managed properly, type 1 diabetes can lead to increased risk of premature death, can have potentially devastating consequences for patients’ health and quality of life, and can affect the quality of life of family members [5-8].

Education is considered an essential part of diabetes care. This is because patients have responsibility for day-to-day management of their diabetes; hence, it is critical that they understand the disease and how to self-manage it effectively [7]. The provision of structured education programmes for people with diabetes is now a requirement in the UK and other countries [9-11]. Such programmes draw on theories of empowerment, goal-setting and problem-based learning and usually consist of group-based learning opportunities where participants engage in practical self-management experiences to facilitate health-promoting behaviours [12]. Similar techniques have been applied in structured education programmes for patients with other long-term conditions, such as chronic kidney disease [13] and chronic obstructive pulmonary disease [14]. Most programmes for type 1 diabetes patients also offer education and instruction in the use of flexible intensive insulin therapy, comprising long-acting basal or background insulin injected once or twice daily, and quick-acting bolus insulin adjusted to carbohydrate intake at meals. This regimen was originally pioneered and used in the Diabetes Treatment and Teaching programme in Germany [15,16] and it now widely advocated and used in type 1 diabetes management. It is taught as part of many structured education programmes, including the Dose Adjustment for Normal Eating (DAFNE) programme in the UK [17,18]. This course is provided free at the point of delivery, is often funded by the National Health Service (NHS), and has been taught to over 23,000 patients in the UK [19]. The DAFNE programme has also been rolled out to countries, including Ireland, Australia, Kuwait, New Zealand and Singapore [11].

In common with other similar programmes, patients who attend a DAFNE course are taught how to count carbohydrates and, using ratios, to calculate quick-acting insulin dosages relative to the amounts of carbohydrate ingested. Patients’ calculations also take account of pre-meal self-monitoring of blood glucose readings and lifestyle factors (e.g. physical activity), which also affect blood glucose levels [15,16]. Patients are encouraged to maintain blood glucose readings within specific target ranges. To do this, they are instructed how to examine/interpret diary records of their blood glucose readings to ascertain whether discrepant patterns emerge and, if apparent, to make changes to background insulin doses and/or quick-acting ratios. While, typically, patients make some changes to background insulin doses and quick-acting ratios during the education programme attended [20], they are encouraged, when necessary, to make further alterations over time. Patients attend over five consecutive days and are usually taught in groups of six to eight by a diabetes specialist nurse and a dietitian.

On completion of their courses, patients in the UK receive routine clinical care, either in hospital or general practice, provided by health care professionals from whom they received clinical care and reviews prior to attending DAFNE. In addition, patients are provided with educators’ contact details and invited to call or email when necessary if they have any questions or concerns. Patients are also invited to return to their DAFNE centres to attend a half-day, group-based follow-up session six weeks post-course facilitated by the same educators and involving their fellow course attendees. These sessions take place either in the morning or afternoon on a fixed date chosen to ensure educators’ availability. During these sessions, patients are asked to present records of recent blood glucose readings for review, discuss any issues which have caused concern, and share and compare experiences with fellow attendees. Some DAFNE centres also offer similar group, peer-based follow-up sessions at six and/or 12 months.

Outcomes are fairly consistent for patients attending DAFNE and similar structured education programmes in different countries, with attendees experiencing short-term (six month) improvements in glycaemic control, quality of life and dietary freedom [15,17,21], and reductions in incidence of severe hypoglycaemia [22,23]. However, while improvements in quality of life are maintained, patients experience a decline in their glycaemic control over time, indicating that they have not fully sustained the self-care practices taught on their courses [24,25]. Similar trends have been identified among graduates of programmes for patients with type 2 diabetes [12,26]. The reasons for this glycaemic drift are poorly understood and, it has been suggested, require in-depth investigation of patients’ own understandings and experiences after attending structured education programmes [18] and research to determine better ways to support them [25].

Despite the widespread adoption of structured education, little is known about patients’ views, and preferences for, clinical support post-course and over time, to help maintain improvements in blood glucose control and sustain self-care practices. In addition, reviews have highlighted the disparate and inconsistent provision of follow-up support currently on offer [27-29]. While an investigation of group-based follow-up for type 1 diabetes patients is underway [30], our study was designed to explore what support DAFNE graduates consider most helpful to sustain self-care practices and improvements in biomedical outcomes over time. To our knowledge, this is the first study to explore the support needs of patients after attending structured education providing instruction in flexible intensive insulin therapy, in depth and over time, and forms part of a broader longitudinal investigation of patients’ self-care practices post-course [31,32]. Our aim, in the research which forms the focus of this article, was to explore: patients’ experiences of, and views about, their health care and the support they currently receive post-course; unmet support needs; and, their recommendations for future health care support. Our objective was to inform development of future support provisions for patients following participation in structured education programmes such as DAFNE.

## Methods

### Design and study sample

A qualitative longitudinal design was employed in which patients were interviewed immediately after attending a DAFNE course and six and 12 months later. This design enabled patients’ (changing) support needs and likes/dislikes of existing support provision to be captured and explored in-depth and over time [33]. Interviews also afforded the flexibility needed for patients to raise issues they perceived as salient, including those not anticipated at the study’s outset [34].

Participants were recruited from six DAFNE courses hosted in five DAFNE centres across the UK. These included well-established DAFNE centres (where structured education had been offered for some time) and relatively new centres (where a smaller number of staff had delivered fewer courses). Patients who were due to attend one of these courses were approached by their course educators to explore whether they would be willing to take part in the interview study. A minority of patients (n = 5) did not wish to participate and, when asked about their decision, cited practical reasons such as being unavailable to take part in follow-up interviews. The majority of patients who expressed willingness to participate were then contacted by a team member to discuss the study, prior to giving written informed consent to take part. Thirty adult patients were recruited into the study, as this sample size allowed a full range of themes and issues to be identified and explored in-depth and for data saturation to occur (data saturation happens when no new findings or themes arise from an analysis of new data collected) [35,36].

The last two courses were purposively sampled to ensure diversity of gender, age and duration of diabetes in the final sample, and so that the demographic profile of the people who took part in the study were similar to that of DAFNE attendees as a whole. Patients were not sampled on the basis of their ethnicity as, at present, only a minority of DAFNE attendees belong to minority ethnic groups. Sample demographic and biomedical data are presented in Table 1. Recruitment and the first round of interviews were staggered to permit concurrent data collection and analysis, in line with an inductive approach. This enabled issues and themes identified during early phases of data collection to be examined in greater depth in later phases [37].

**Table 1:** Demographic characteristics, glycaemic indicators and participation rates of 30 adult patients (values are expressed as mean ± SD or percentage)


Age (years)	36.1 ± 11.6; range 18–56	
Gender	16 (53.3%) female	
Diabetes duration at recruitment (years)	16.5 ± 10.3; range 1–45	
HbA_1c_ - baseline (%; mmol/mol)	8.8 ± 1.9, range 5.4 – 12.7; 73 ± 20, range 36 – 115	
- 12 months (%; mmol/mol)	8.2 ± 2.0, range 6.0 – 14.1; 66 ± 22, range 42 – 131	
Occupation (no. and% at recruitment)		
- professional (health, education etc.)	9 (30%)	
- semi-skilled	11 (36.7%)	
- unskilled	6 (20%)	
- student	3 (10%)	
- unemployed	1 (3.3%)	
Interview participation at:		
- First round	30 (100%)	
- 6 months	28 (93.3%)	
- 12 months	27 (90%)	

The study was approved by the National Research Ethics Service: King’s College Hospital Research Ethics Committee (ref: 08/H0808/53).

### Data collection

Semi-structured interviews were conducted with patients between July 2008 and February 2011. The first round of interviews mainly took place face-to-face and in patients’ homes, in the week following DAFNE course attendance, with follow-up interviews conducted by telephone. Comparison of patients’ accounts showed that the setting did not influence responses, while face-to-face contact during the first round of interviews helped develop a rapport to facilitate follow-up data collection [38]. Interviews were informed by topic guides, developed following literature reviews, observations of courses [20], and inputs from health care professionals, and were revised in light of ongoing data analysis. The first round of interviews explored patients’ accounts of support received prior to the course and the support they envisaged might be necessary to sustain effective use of a flexible intensive insulin regimen.

Concurrent analysis of data, and repeated reading of previous rounds of interview transcripts by study team members, informed the development of topic guides used at six and 12 month follow-ups. These follow-up interviews examined: patients’ perceptions of support sought and received post-course; their views on future support requirements; and, their unmet support needs. Relevant areas explored during the study included:

- patients' views about pre-course support they had required, and who this was provided by, since diagnosis, and over time.

- patients' views on their post-course support needs, including: what they had found helpful and unhelpful over time; and, patients' experiences of attending structured follow-up meetings in diabetes centres.

- patients' experiences of receiving support from health care professionals, including at routine follow-up appointments

- patients' views on post-course encounters/contacts with fellow course attendees; and whether, and how, patients' training impacted on subsequent encounters with health care professionals

- patients' views about unmet needs necessary to help sustain flexible insulin therapy over the longer-term

- patients' views about support sought from and offered by family/friends and work colleagues, whether this has changed over time and whether there have been any changes to ways in which family/friends/colleagues are involved.

While the same topics and areas were explored with all patients in the follow-up interviews, supplementary questions were included which were personalised for each patient based on a review of their earlier interview(s). For example, if a patient at baseline had indicated their intention to seek support or advice from a course educator, the six month follow-up interview included questions which explored whether and how this had affected their diabetes management and if the person had required further support. All interviews were audio-recorded, averaged 60 minutes, and were transcribed in full.

### Data analysis

The method of constant comparison [37] was used to develop a framework of themes for coding and further analysing the data. To achieve this, patients’ interview transcripts were read and re-read sequentially by DR and JL, who each undertook an independent analysis to compare and contrast patients’ accounts. The data were also examined for contradictory evidence to counteract the possibility of researcher bias. Regular team meetings were held to compare interpretations, resolve any differences in understanding and to reach consensus on recurrent themes [39]. A coding framework was devised after agreement on key themes was reached. Data were coded by DR using a qualitative software programme (QSR N6; QSR International, Doncaster, Australia), which facilitated additional coding and data retrieval to permit further examination of specific datasets. A subset of transcripts was independently coded by JL to ensure consistency and any disagreements were resolved by discussion. Very few anomalies in interpretation were noted. Data collection stopped when no new themes arose from the data. Below, data are tagged with participants’ gender, identifying number and interview round (e.g. F3.2 refers to the second (six month) interview of female participant 3).

## Results

### Contextualising the findings

In their immediate, post-course interviews, most patients reported liking and feeling motivated to sustain flexible intensive insulin therapy rather than reverting to former treatments, such as those comprising fixed insulin doses. Patients with a history of poor glycaemic control, such as F4, explained how this motivation arose from a perception that flexible intensive insulin therapy offered a more “logical process” for managing her diabetes than her previous regimen comprising two fixed daily doses of insulin. Continuing, F4 explained how the new approach enabled her to self-manage blood glucose levels precisely: “it’s all very formulaic and I like that, being given a formula and dealing with it” (F4.1). However, due to its technical complexity, most patients anticipated, or were already encountering, difficulties applying this regimen in their everyday lives, especially when deciding if changes to quick-acting ratios or background insulin doses were needed. For example, while praising the support offered by educators during her DAFNE course, F7 went on to express concerns about making independent adjustments without their continued input and oversight: “I’m slightly unsure, you know, now I’m basically on my own and I’ve reduced the background insulin and it’s slightly ‘ooooh, I’m not sure I should be doing that” (F7.1).

To address the technical difficulties of adjusting ratios and background doses, and to sustain effective use of flexible intensive insulin therapy, most patients, in both their post course and follow-up interviews talked predominantly about needing and/or valuing input from health care professionals. Support needed or received from family and friends featured much less prominently in their accounts. Hence, the findings reported below focus on health care professionals’ support. As patients’ accounts of clinical support needs at six and 12 months were very similar, this is reflected in the presentation of data below.

### Seeking reassurance and trouble-shooting opportunities

Follow-up interviews revealed multiple instances where patients had sought out and consulted their course educators to seek affirmation and reassurance that they had followed the correct course of action. F3, for instance, who switched from using fixed doses of insulin, described “playing around with the ratios” in response to high blood glucose readings following her course, but subsequently discussed her actions with the educator: “just so that I know I’m going in the right direction” (F3.2).

Most typically, patients were apprehensive about whether they had interpreted patterns in blood glucose readings correctly and determined the correct course of action. These concerns led several patients to contact their course educators prior to making a change to treatment. For example, F7, who had earlier expressed concerns about making independent adjustments to insulin (see above), reported how, by the time of her second round interview, she had sought advice from educators before taking action to address high blood glucose readings: “I double-checked that I wasn’t having too little of the background insulin. I just wanted to make sure” (F7.2). Others, such as F4, who became worried about recurring fluctuations in blood glucose readings, also solicited educator input and support because she was uncertain how to address this problem on her own: “I’ve sort of said, ‘should I put my background up one [unit], it’s going high here?’ And she’s [educator] like, ‘well, give it until the end of the week, you know, let your body settle down a little, but don’t make changes too quickly” (F4.2).

### Experiences of seeking support from health-care professionals

#### Health care professionals with relevant training

When seeking support, virtually all patients, as the above data suggest, expressed strong preferences for consulting their course educators, who many, post-course, now considered their first “port of call” (F6.2). Patients’ decisions to approach course educators were often informed by post-course experiences of having obtained unsatisfactory support from alternative sources. This included nurses who had not received relevant training or general practice staff from whom some patients received their routine clinical reviews and who, it was claimed: “haven’t heard of DAFNE” (F3.2) or “don’t know the subject… they’re not specialist” (M12.3). M4, for instance, who attended routine appointments at his local GP surgery, described how his practice nurse had told him soon after the course that he now knew “more about it [diabetes] than she does, which doesn’t inspire me with loads of confidence”. Dissatisfied with this experience, M4 concluded: “if you have a problem you might as well take it into [the diabetes centre at the hospital], even though it’s a long way to go and a bit inconvenient” (M4.2).

#### Lack of support at routine hospital appointments

At follow-up, several patients recounted how their routine hospital appointments were conducted by staff without DAFNE training and offered what they now considered nominal levels of support. Specifically, such patients reported how routine clinical review appointments tended to focus on HbA_1c_ readings, (which is a measure of average blood glucose levels over previous months), whereas, post-course, patients now wanted, and felt they needed, a more holistic appraisal from health care professionals, which included a review of daily blood glucose readings. For example, F13 described how obtaining a HbA_1c_ result considered satisfactory by her clinician had led to a cursory clinical review and had restricted opportunities to raise concerns about day-to-day fluctuations in blood glucose readings: “I think she [consultant] maybe thought because of my Hb[A_1c_] that everything was fine and that I didn’t really need to speak to her, so it kind of put me off” (F13.2).

Patients’ post-course critiques of clinical support were also informed by revisions made to their understanding of diabetes, which, coupled with an enhanced sense of empowerment, affected the dynamics of clinical consultations. For example, having participated in a DAFNE course, M9 explained how he had become: “more demanding [of health care professionals], just because I know a bit more about it now and I feel a lot more in control”. Attending an annual review and concerned that he had not been questioned in more detail about his blood glucose readings, M9 reported how: “I’m putting all this bloody effort in, can’t they do the same, they’re not taking much of an interest” (M9.3).

#### Accessing educators

While all patients had access to course educators’ telephone and email contact details, some found that calls or requests for advice left on answer-machines were not returned. This, as M9 explained, was off-putting and led to his decision to only contact educators with “really important issues” because “I don’t want to bother them unless I feel that I need to” (M9.2). More often, patients expressed a reluctance to initiate contact with clinicians/educators in-between scheduled appointments (e.g. clinical reviews), despite having questions and concerns. This included F16 who struggled with high readings but waited until a scheduled review appointment before seeking advice as she did not wish to present herself as a burden: “it’s one of my, just personal traits, is I find it very hard to ask people for help in most things” (F16.2).

In contrast to patients who struggled to approach educators, others described the benefits of seeking out and receiving technical advice and individualised trouble-shooting support from professionals with relevant training. For example, when seeking an explanation for high blood glucose readings, and noting that it was “down to me” to initiate contact, M12 began regular email exchanges with course educators who reviewed electronic records of his blood glucose readings “a couple of weeks at a time.” Furthermore, he described having made several changes to his insulin doses after receiving advice from educators during these communications: “I was doing a little bit of exercise and I was talking to [the educators] about what was happening with the readings and they suggested I should try to reduce it [background insulin]” (M12.2).

### Organised follow-up meetings

#### Attendance at group-based follow-up

While educators provided graduates with a fixed date and time to attend a follow-up session at the end of their course, less than half of the patients attended the six week event and fewer still subsequent meetings. In several instances, patients implicated practical difficulties: “something to do with work got in the way” (M2.2) or had family commitments which took precedence. Likewise, F10, who worked full-time in a busy, professional role, described how a lack of flexibility in the time-tabling of follow-up events had made it difficult for her to attend: “I didn’t go to the six month event… I just kind of, eh, was slightly busy at work, knew that I had my annual review coming up anyway… and retinopathy screening and I thought, I’m not asking for any more afternoons off work to go to the hospital” (F10.3).

Several patients suggested that low rates of participation at six week follow-ups had limited the opportunities to replicate the dynamics/synergies of initial courses. After attending a follow-up with one other patient present, F8 explained: “it makes for a very different session [at follow-up] because a lot of those DAFNE sessions [on courses] are actually sharing experiences” (F8.2). Yet, despite small group sizes, patients who attended follow-ups described having derived several benefits. These included: opportunities to confirm others’ successful application of the DAFNE approach and observe “the improvement that people had [made] in six weeks” (F2.3); being able to discuss “common themes” (M11.2) and seek solace from others: “it was reassuring to find out that other people are having the same problems as you are… Christmas sucked” (M9.2); and, opportunities to counter isolation by speaking with fellow patients who: “know what planet I’m from… if I’m talking in diabetes language” (F3.2). Several patients also described how follow-up sessions were intrinsically motivational and stimulated renewed attention to regimen principles: “it [DAFNE] gave me the confidence to think, actually I can control this. … but I think follow-ups, just sort of re-confirm people’s confidence” (M5.2).

#### Limitations of group-based follow-up

As documented elsewhere [13] the group-based approach employed on the original five day DAFNE courses helped instil the knowledge and skills necessary to convert to flexible intensive insulin therapy, and was well received by virtually all patients. However, while patients, in their follow-up interviews, highlighted some benefits to be gained from attending follow-up sessions in a group, most indicated a preference and need for one-to-one support. This included M7, who described group-based follow-ups as mitigating opportunities for patients to: “talk about their own individual circumstances … everyone’s an individual and I think everyone has individual needs… and events happening in their lives” (M7.3).

Several patients also expressed dissatisfaction with reviews of blood glucose readings at six week follow-up sessions. While patients had collected blood glucose data for six weeks, the requirement for all patients’ readings to be reviewed meant there was only time to examine their most recent results. M14, for instance, described how educators had reviewed blood glucose readings that he had gathered over the preceding two or three days, which, he suggested, could result in a focus applied to an unrepresentative sample of results collected over “a very small period of that six weeks”. To allow for a more detailed examination of blood glucose readings collected over the six week time-frame, M14 suggested that he would have benefited from: “a bit of a one-to-one with [the educators] so they could have a look through your diary, sort of page to page, just have a look to see maybe if they’ve spotted something that I hadn’t spotted.” (M14.2).

In other accounts, patients sought and/or expressed a preference for individualised and tailored support, provided by specialists, that was responsive to changes in their personal circumstances and lifestyles. For example, F2 described having needed, and received, regular and intensive educator support after she became pregnant, to review and change quick-acting ratios and basal insulin doses, to control unstable and fluctuating blood glucose readings. Other patients’ need for specialist, individualised support also followed significant changes in life circumstances, which, in turn, affected their insulin requirements, in ways which were specific to themselves. For example, discussing his decision to “up the ante a little bit” in the month after attending the course M12 explained that he had contacted educators for support to adjust insulin to counter an increase in physical activity after moving from a sedentary role to one involving manual labour: “I’ve spent a year working out in the field… and my dosages changed big time, my diet changed big time” (M12.3). Highlighting an on-going need for support, this patient later reported how a subsequent injury had limited his physical activity, which necessitated further advice from educators about insulin adjustments.

### Provisions to address unmet needs

To address their needs for individualised support and trouble-shooting opportunities, some patients suggested that it would be beneficial to have a dedicated “emergency line” (M8.1) or “twenty-four hour helpline” (F7.1), staffed by clinicians with relevant training: “[I’d] like the ability to … maybe phone up the diabetic nurses, the ones who are DAFNE-trained, and knock some things about if I had a question” (M13.1). While identifying this need for readily-accessible support, patients also conveyed concerns about placing additional burdens on clinical staff who, many felt, already had substantial workloads. Hence patients suggested that future support services should be delivered by dedicated staff: “perhaps somebody who was just 100% DAFNE, rather than having to split themselves into two or three different positions … and to know that you’re not going to be interrupting anybody’s clinics” (F6.3). Several patients also discussed how a “structured appointment system,” put in place post-course, might address concerns about unduly “bothering them [educators]” (F4.3). This system, it was suggested, could help patients who were reticent about making impromptu contact with educators. For example, F16, who, as described earlier, worried about burdening educators with her questions and concerns, felt that an appointment system: “would make it easier to approach them rather than being left to just approach as an individual” (F16.3).

Finally, patients described how the provision of “refresher courses” (M6.3), delivered by health care professionals, could address several needs, including: opportunities to access top-up or new information; and, re-education to aid patients’ recall and use of information that had not been well retained (e.g. the effects of sickness or physical activity on blood glucose control).

## Discussion

This is the first qualitative study to explore in depth and over time type 1 diabetes patients’ views about, and need for, health professional and health service support after attending a structured education programme promoting flexible intensive insulin therapy. The findings reported here lend empirical support to international guidelines and studies [9,29,40] which recommend providing continuing support to graduates of diabetes self-management education programmes. This study also offers insights into what forms of long-term support health care providers could consider making available to patients to best meet their own preferences, personal circumstances and perceived needs. Patients highlighted a preference and need for individualised and tailored inputs to accommodate their unique and personal experiences of self-management and of applying their insulin regimens in their everyday lives. Patients also expressed a need for this support to be ongoing and provided by professionals who had received training in the principles and practices taught on their course. While patients highlighted benefits to attending group-based follow-ups, principally to receive empathy and emotional support from peers, they described needing tailored, professional inputs post-course to optimise and sustain glycaemic control, and trouble-shoot issues of concern. Furthermore, patients highlighted a need for support which could be accessed as and when it was required and without them feeling they were burdening staff. To address unmet needs, patients suggested that readily accessible forms of support, such as a telephone helpline, could be provided by dedicated professionals trained to support their self-management approaches.

Peer support, often provided in a group-based setting, has increasingly been recommended as a potential model for the provision of diabetes self-management support [41]. However, the clinical benefits of group-based support have recently been called into question in the findings from a RCT, conducted by Smith et al. [42]. Consistent with findings of other studies of group-based peer support for type 2 diabetes patients, this RCT failed to show a significant impact on glycaemic control. Poor attendance at group meetings was also observed. Hence, these authors recommend that further research, including work focusing on alternative models of delivering support, be conducted. Our qualitative study adds to Smith et al’s concerns about promoting group-based support in diabetes clinical practice, particularly if this support is offered in isolation from other types of inputs and interventions. We have also provided insights into why group-based follow-ups may not necessarily be a popular or effective approach – albeit in this instance, through a focus on type 1 diabetes patients. Specifically, we have shown that a group-based approach may be incompatible with patients’ need for individualised input from health professionals post-course, to accommodate their specific and personal experiences of applying their treatment regimens in everyday life. In addition, prescheduled, group-based appointments, selected to ensure educators’ availability, were found to be incompatible with patients’ need to access health professional input as and when it was required. Although follow-up sessions enabled some patients to meet together, this format was deemed inflexible by those who had to juggle attending appointments with work and family commitments.

Like the type 2 diabetes patients Cooper et al. interviewed after attending a structured education programme [43], our study participants encountered frustrations and difficulties when post-course care and reviews were provided by professionals who lacked specialist knowledge and training in course practices and principles. As Cooper et al. suggest, not only are such professionals unable to provide the specific clinical input graduates may require to sustain the self-management approach taught on the course, they may also be unwilling to embrace, and work in partnership with patients who, by virtue of course attendance, feel more knowledgeable and empowered. Hence, Cooper et al. recommend that follow-up care be provided by staff trained in course principles and practices. Our findings lend support to Cooper et al. and, indeed, we would suggest that their recommendation to use appropriately trained staff is extended to graduates of structured education programmes for type 1 diabetes.

In addition, our findings suggest that patients attending programmes such as DAFNE may potentially benefit from the provision of a ‘menu’ of support options post-course and over time. Such a menu could incorporate differing degrees of professional inputs tailored to patients’ personal requirements, and which are responsive to changes in lifestyle. To this end, consideration in a future research study or pilot intervention could be given to suggestions made by our study participants, including provision of telephone- and/or email-based support and counselling, delivered by trained professionals. Such interventions have not yet been investigated in the context of follow-up care provided to graduates of structured education programmes for type 1 diabetes. However, they have been explored and promising results found in other studies which offer support to improve healthy eating, weight loss and self-management behaviours for patients with type 2 diabetes. In these studies, telephone- and email-based support have been shown to promote: maintenance of health behaviour change [44,45]; increased use of self-care behaviours [46,47]; and, positive impacts on glycaemic control [48]. Such interventions have also been found to enable patient-centred approaches where clinicians’ information/advice can be customised to accommodate individuals’ life circumstances [49,50] which, in turn, enhance self-care behaviours [51].

When piloting, developing and/or evaluating interventions to improve follow-up support for structured education programmes delivered to patients with type 1 diabetes, attention could also be usefully given to the ways in which traditional forms of service delivery can shape patients’ expectations and behaviour [52]. For example, patients in our study did worry about burdening clinicians with their questions and concerns, and/or did not consider that they had the right to do this. Hence, some patients either did not ask for help at all or they withheld presenting their concerns until they could do so opportunistically at a scheduled review appointment, which could result in considerable time elapsing before a problem was resolved. To address patients’ concerns about over-burdening staff, follow-up support may thus need to be presented as an integral part of their care-package and, hence, as something that they can expect and feel entitled to ask for. Health care professionals might also consider initiating telephone contact with patients to establish their progress and support needs. In addition, provision of a dedicated phone or email service staffed by clinicians employed specifically for this purpose, may help overcome patients’ anxieties about burdening staff and enable them to seek help when questions and problems arise. Finally, our findings highlight a divergence between patients’ post-course expectations of clinical reviews and their need for detailed examination of blood glucose readings, and the emphasis clinicians placed on HbA_1c_ readings. To address patients’ needs for specific advice on insulin dose adjustment, it may be necessary for staff delivering clinical reviews to explore reasons which underpin individuals’ day-to-day fluctuations in blood glucose readings.

### Strengths and weaknesses

A key strength of this study is that the 12 month follow-up time-frame permitted longitudinal exploration of the types of support patients anticipated needing post-course, their likes and dislikes of support actually received, and identification of unmet support needs. However, as indicated above, patients’ support needs were similar at both six and 12 month follow-up. Hence, in future studies, researchers may wish to explore these issues at an earlier point in time following course completion. In terms of study limitations, it should be noted that as patients opted in (this being a requirement stipulated by the research ethics committee), they may have been more motivated to implement treatment practices taught on the course and/or more critical of existing care provisions. Furthermore, while some key findings resonate with those from studies involving type 2 diabetes patients [42,43], our study was restricted to type 1 diabetes patients, and, more specifically, to those converted to flexible intensive insulin therapy. While patients in our study were required to adjust their own insulin doses, patients with other long-term conditions usually are only asked to make changes to lifestyle; hence the types of support necessary may differ between long-term conditions. To assess the generalisability of our findings, further work could be undertaken with graduates of other types of programme, and with other conditions, to determine whether our patients’ accounts of support needs are generalisable or specific to particular disease states and treatments. Future work could be undertaken to compare different approaches to follow-up that addresses the unmet needs identified in our study and which complements existing group-based provision. Further work would also be necessary to obtain health care providers’ views to explore how the approaches highlighted above could be operationalised most effectively.

## Conclusions

This is the first study to explore in depth and over time type 1 diabetes patients’ experiences of, and perceived need for, follow-up support following participation in structured education. Our findings identify potential limitations to group-based forms of follow-up support, and highlight alternative types of support which might better meet patients’ preferences, circumstances and needs. To help maintain the self-care practices taught on their courses, patients may benefit from being offered and having access to support provided by health care professionals with relevant training. Patients may also benefit from support which is tailored and personalised to enable them to apply their education and training in their everyday lives.

## Competing interests

SRH has undertaken consultancy for NovoNordisk, Eli Lilly & Abbott for which his institution has received payment. He has given talks on behalf of NovoNordisk, Eli Lilly & Sanofi Aventis for which he has received payment. He is Chief Investigator on a trial measuring the benefits of CSII in type 1 diabetes funded by NIHR Health Technology Assessment. The remaining authors declare that they have no competing interests.

## Authors’ contributions

DR collected data, performed data analysis and interpretation and drafted the manuscript. JL designed the study, performed data analysis and interpretation, contributed to the discussion, and reviewed and edited the manuscript. DDC, SRH and JE contributed to the discussion, and reviewed and edited the manuscript. All authors read and approved the final manuscript.

## Pre-publication history

The pre-publication history for this paper can be accessed here:

http://www.biomedcentral.com/1471-2458/12/652/prepub
